# Rare Earth Elements and Bioavailability in Northern and Southern Central Red Sea Mangroves, Saudi Arabia

**DOI:** 10.3390/molecules27144335

**Published:** 2022-07-06

**Authors:** Mohammed Othman Aljahdali, Abdullahi Bala Alhassan

**Affiliations:** 1 Department of Biological Sciences, Faculty of Science, King Abdulaziz University, P.O. Box 80203, Jeddah 21589, Saudi Arabia; 2 Department of Biology, Faculty of Life Sciences, Ahmadu Bello University, Zaria 810001, Nigeria

**Keywords:** mangrove, rare earth elements, distribution, bioaccumulation, *Avicennia marina*, Red Sea

## Abstract

Different hypotheses have been tested about the fractionation and bioavailability of rare earth elements (REE) in mangrove ecosystems. Rare earth elements and bioavailability in the mangrove ecosystem have been of significant concern and are recognized globally as emerging pollutants. Bioavailability and fractionation of rare earth elements were assessed in Jazan and AlWajah mangrove ecosystems. Comparisons between rare earth elements, multi-elemental ratios, geo-accumulation index (Igeo), and bio-concentration factor (BCF) for the two mangroves and the influence of sediment grain size types on concentrations of rare earth elements were carried out. A substantial difference in mean concentrations (mg/kg) of REE (La, Ce, Pr, Nd, Sm, Eu, Gd, Tb, Dy, Ho, Er, Tm, Yb, and Lu) was established, except for mean concentrations of Eu, Gd, Tb, Tm, and Lu. In addition, concentrations of REEs were higher in the Jazan mangrove ecosystem. However, REE composition in the two mangroves was dominated by the lighter REE (LREE and MREE), and formed the major contribution to the total sum of REE at 10.2–78.4%, which was greater than the HREE contribution of 11.3–12.9%. The Post Archean Australian Shale (PAAS) normalized values revealed that lighter REE (LREE and MREE) were steadily enriched above heavy REE. More so, low and negative values of R_(H/M)_ were recorded in the Al Wajah mangrove, indicating higher HREE depletion there. The values of BCF for REEs were less than 1 for all the REEs determined; the recorded BCF for Lu (0.33) and Tm (0.32) were the highest, while the lowest BCF recorded was for Nd (0.09). There is a need for periodic monitoring of REE concentrations in the mangroves to keep track of the sources of this metal contamination and develop conservation and control strategies for these important ecosystems.

## 1. Introduction

The Red Sea is a channel that forms a linkage between the Mediterranean Sea (north) and the Indian Ocean (south). The sea is a marine biodiversity hotspot with a high abundance of coral reefs, mangroves, and sea grass [1,2]. In aquatic ecosystems such as the Red Sea, suspended sediments and particulate matter may account for almost 90% of metal burden [3].

Rare earth elements (REEs) are a collection of seventeen chemical elements in the periodic table and are generally trivalent elements, excluding Ce and Eu, which tend to exist as Ce (IV) and Eu (II). REEs starting from La and ending with Sm are considered light rare earth elements (LREEs), while those ranging between Gd and Lu are considered heavy rare earth elements (HREEs) [4]. The light rare earth elements (LREEs) and heavy rare earth elements (HREEs) have analogous geochemical behaviors. They give a better understanding of complex procedures of a geochemical nature that single proxies cannot readily discriminate due to their coherent and expectable characteristics [4,5].

REEs do not occur in pure metal form even though they occur in nature, although Promethium, the rarest, only occurs in trace quantities in natural materials as it has no stable or long-term isotopes [6]. Globally, REEs are recognized as emerging micro-pollutants in aquatic ecosystems [7,8]. Modern technologies, on the other hand, utilize REE for its unique physicochemical properties in high-tech applications [9]. For example, AgInSe_2_ (AIS) is one of the most attractive materials in thin film solar cell applications because of its high optical absorption coefficient [10]. As a result, it is unlikely that REE would spread naturally in most coastal habitats such as mangroves because they have already been impacted by anthropogenic activities [8,11,12].

Mangroves are important intertidal coastal systems that provide multiple ecological functions. They regulate material exchange at the interfaces between land, atmosphere, and marine ecosystems [13,14]. Mangrove ecosystems are dynamic in nature, often subjected to rapid changes in physicochemical properties such as water content, pH, salinity, texture, and redox conditions due to tidal flushing, and the associated flooding could influence metal contamination. Flooding may develop redox cycles in the aquatic environment, with alternating periods of oxidizing and reducing conditions [12,15,16,17]. Therefore, the application of REE can be useful in tracing the channels and processes in which these elements are involved, particularly in contaminated environments such as those found in the Jazan and AlWajah mangrove ecosystems and their biota.

Notably, there are few or no studies providing a comprehensive investigation of the bioavailability of rare earth elements in the mangrove ecosystems of Jazan and AlWajah in the northern and southern central Red Sea. As a result, this study will open the way for periodic monitoring of REE concentrations in mangrove ecosystems, as well as tracking the sources of metal contamination, allowing for the development of policies for the control and conservation of these important ecosystems.

## 2. Results

### 2.1. REE Composition in Sediment and Influence of Grain Sizes

In this study, the results for REE composition in sediment showed significant variation (*t*-test, *p* < 0.05) between the two mangrove ecosystems investigated (Table 1). A substantial difference in mean concentrations (mg/kg) of REEs (La, Ce, Pr, Nd, Sm, Eu, Gd, Tb, Dy, Ho, Er, Tm, Yb, and Lu) was also recorded; however, except for the mean concentrations of Eu, Gd, Tb, Tm, and Lu, significantly higher concentrations of REEs were recorded in sediment samples collected from the Jazan mangrove ecosystem (Table 1). Generally, the concentrations of REEs were lowest in the Al Wajah mangrove ecosystem. The sum of REEs (∑REE = 112.54 ± 10.48 mg/kg) recorded at the Jazan mangrove was about double that of Al Wajah (∑REE = 78.47 ± 7.89 mg/kg). Nevertheless, the REE composition in the two mangroves was dominated by the lighter REEs (LREE and MREE) and formed the major contribution to the total sum of REEs at 10.2–78.4%, which was greater than the HREE contribution of 11.3–12.9%. In addition, the sum of LREEs (La, Ce, Pr, Nd) was about seven- and eight-fold that of the composition of MREEs (Sm, Eu, Gd) and HREEs (Tb, Dy, Ho, Er, Tm, Yb, Lu), respectively (Table 1).

**Figure 1:**
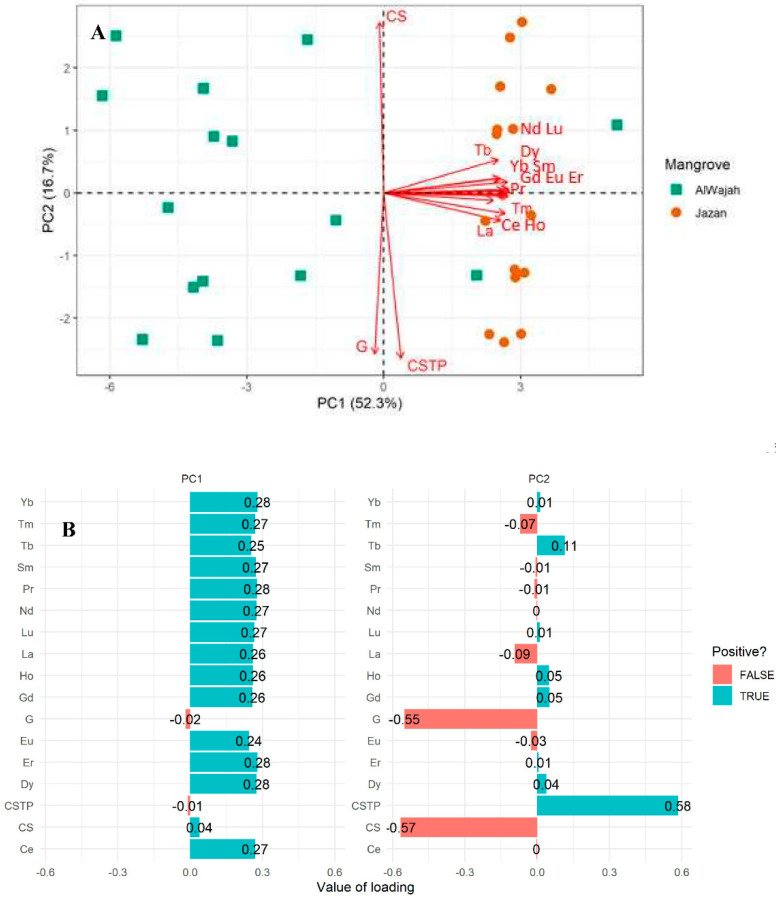
Principal component analysis biplot (**A**) and loadings (**B**) for the relationship between rare earth elements in AlWajah and Jazan mangrove ecosystems. G—Gravel, CSTP—Clay and Silt particles, CS—Coarse sandy.

The principal component analysis biplot revealed the influence of sediment grain size types on REE concentrations in sediment and the site’s contribution to the total variation (Figure 1A). The relationship revealed by the PCA was based on component 1 (52.3%) and component 2 (16.7%), accounting for the total variation of 69.0% (Figure 1A). The coarse sediment correlation grain size and clay silt grain size showed a positive correlation with REEs (Figure 1A,B). The PCA loadings further confirm the positive relationship between the coarse sediment (r = 0.04) and clay silt sediment (r = 0.58) with the REEs to be true (Figure 1B). In addition, clay silt sediment (r = 0.58) has more influence on the concentrations of REEs in the sediment than the other sediment grain sizes; this is revealed to be true in Figure 1B. Relationship analysis based on the site revealed that the Jazan mangrove ecosystem is more influenced by rare earth elements than the Al Wajah mangrove ecosystem.

### 2.2. Fractionation of REE and Sediment Quality Index

**Figure 2:**
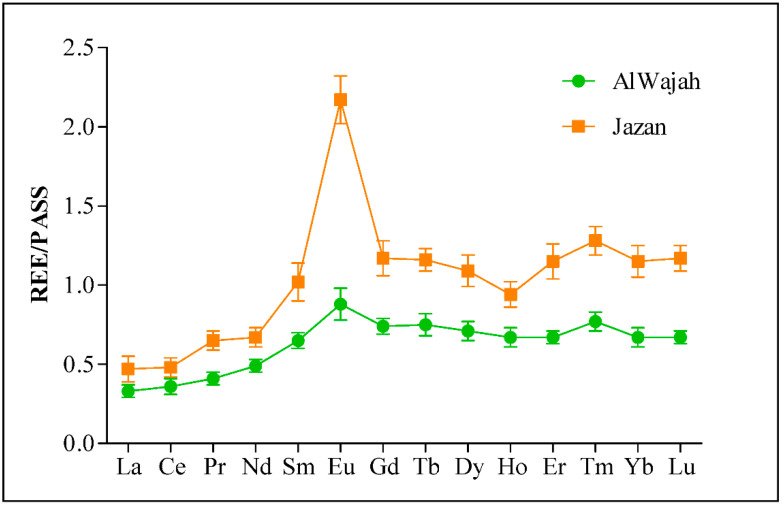
Rare earth elements/Post-Archean Australian Shale (REE/PAAS) patterns for sediments of northern and southern central Red Sea mangrove ecosystems. Values are mean ± standard error.

The Post-Archean Australian Shale (PASS) (Taylor and McLennan, 1985) normalized REE patterns of the sediments for the two mangrove ecosystems plotted provide a better understanding of the pattern of accumulation of REE in this study (Figure 2). The results reveal **∑**REE relative enrichment and comparative trends of fractionation for REE. The fraction (La/Yb)n was higher (0.49) in the Al Wajah mangrove than the Jazan mangrove (0.41), with an average of 0.45 ± 0.04 for the two mangroves (Table 1). For the fractions using (Sm/La)n, a significantly higher value (2.17) was revealed at Jazan, while the lowest value was recorded in Al Wajah sediment samples. The average value for the (Sm/La)n fraction was 2.07, which is the highest median proportion when compared to other fractions, revealing a significant LREE and MREE accumulation (Figure 2; Table 1). The Al Wajah mangrove had the lowest value (1.03) of (Yb/Sm)n, while a significantly higher value (1.13) was recorded for sediment sampled from the Jazan mangrove ecosystem. The average for (Yb/Sm)n in the two mangrove ecosystems was 1.08.

The multi-elemental ratios R(M/L) and R(H/M) indicate positive values corresponding to patterns of fractionation with average MREE enrichment and average HREE depletion. This was supported by the positive range values for R(H/M), the very low positive value for R(H/M) in Jazan, and the negative value at the Al Wajah mangrove, and also a range value from negative to positive (Table 1). There was more enrichment of MREEs at Jazan, with the highest value of R(M/L) (0.28). The low values of R(H/M) and the negative value for Al Wajah indicate HREE depletion, with even more depletion at the Al Wajah mangroves. There exists a significant difference (*t*-test; *p* < 0.05) in the multi-elemental ratios (R(M/L) and R(H/M)) between the two mangrove ecosystems. Ce and Eu anomalies in the two mangroves were revealed by computing the anomalies during the normal and expected shale-normalized REE concentrations, to enable quantification of the probable anomalous concentration. The result revealed a small negative anomaly for Ce, as the average value was almost equal to one. Congruent with the average value, the values of Ce in Al Wajah (0.97) and Jazan (0.86) are still small and negative, with that of Al Wajah being slightly lower. In contrast, the Eu anomaly is small and positive (1.63), with slightly lower (1.27) and higher (1.98) Eu anomaly values at the Al Wajah and Jazan mangroves, respectively (Figure 2; Table 1).

**Figure 3:**
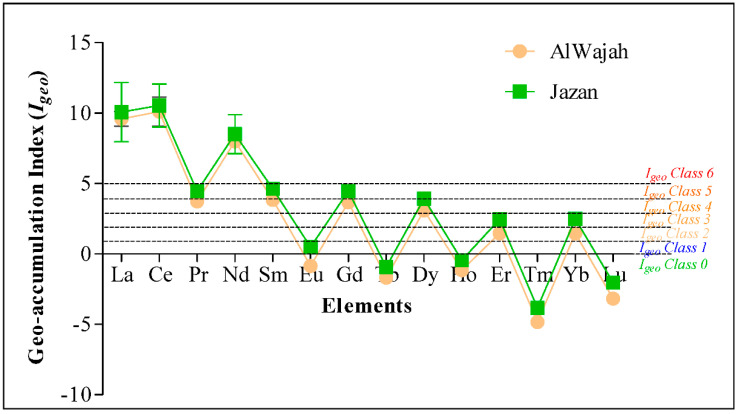
Geo-accumulation index of rare earth elements at Al Wajah and Jazan mangrove ecosystems. Values are mean ± standard error.

The sediment quality index used in this study was the geo-accumulation index (Igeo), which has seven classes of enrichment as described by Muller (1969). Using the Igeo, sediments from the two mangroves were strong to extremely contaminated (4 ≤ Igeo ≥ 5) with La, Ce, Pr, Nd, Sm, and Gd, and moderately to strongly contaminated (1 ≤ Igeo ≥ 3) with Dy, Er, and Yb (Figure 3). In addition, Igeo revealed that the sediment was not contaminated (<0) with Eu, Tb, Ho, Tm, or Lu, with negative Igeo values (Figure 3).

### 2.3. REEs in Mangrove Avicennia Marina and Bio-Concentration Factor (BCF)

**Figure 4:**
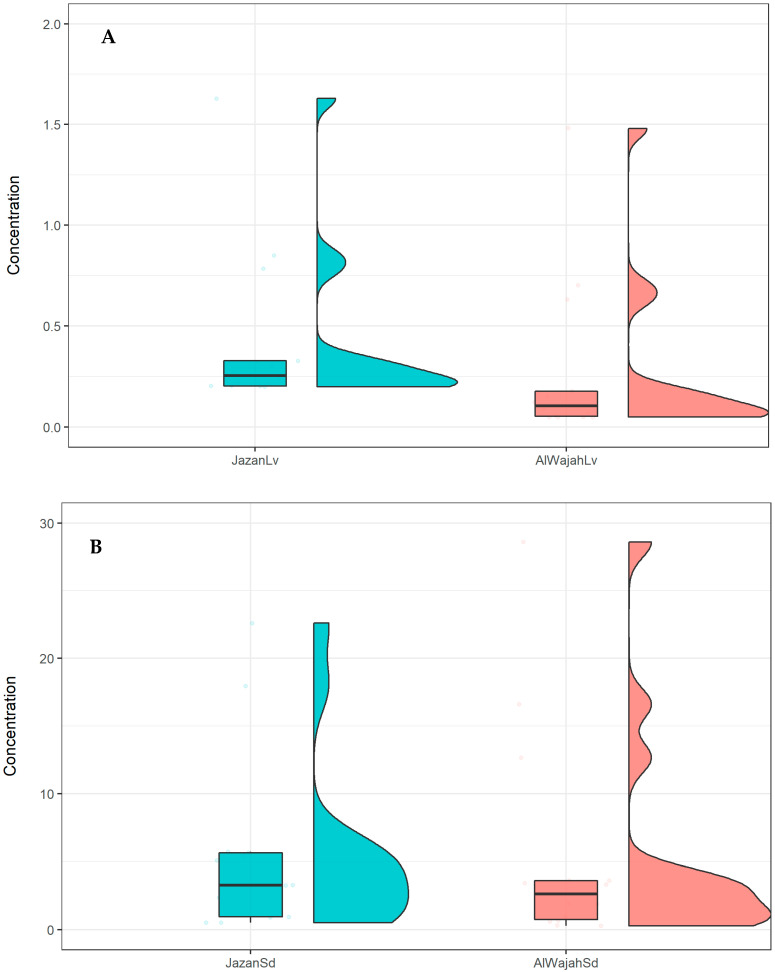
Box and violin plot for distribution of REE in mangrove sediment (**A**) and *A. marina* (**B**). The boxplot for each mangrove displayed the distribution based on the minimum, median, and maximum value, while the violin plot depicts the distribution and density of variables for each mangrove.

**Figure 5:**
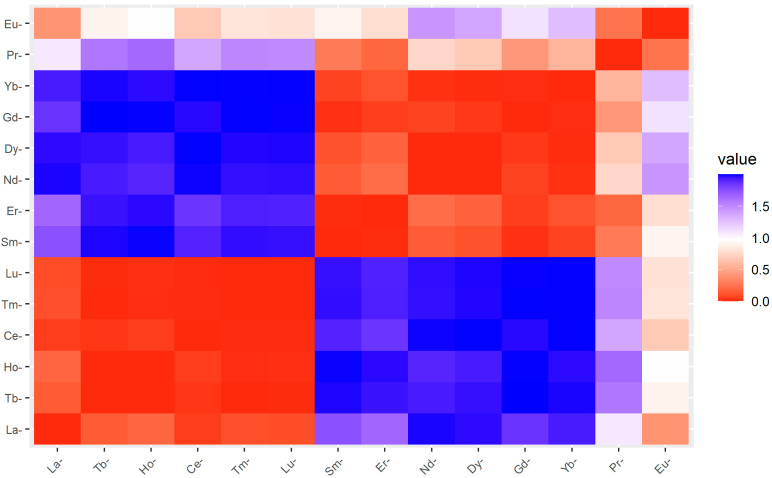
Heat map for the relationship between rare earth elements in sediment and *A. marina*.

**Figure 6:**
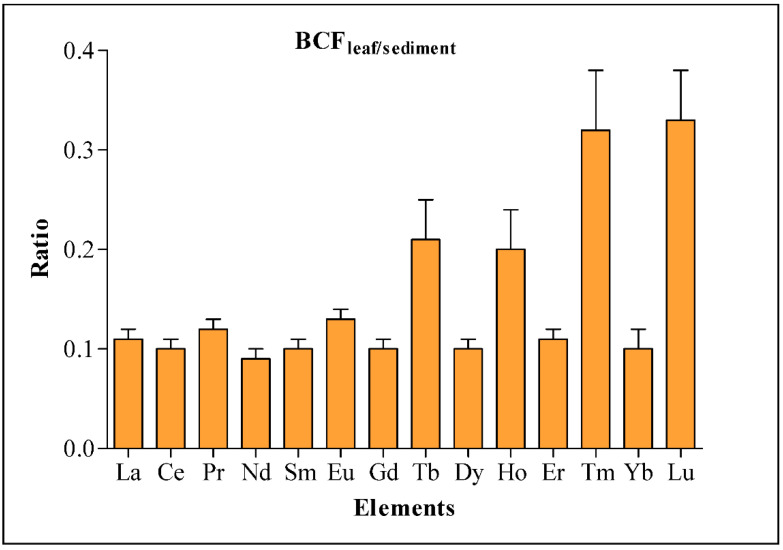
Bio-concentration factor (BCF) for REE composition in northern and southern central Red Sea mangrove ecosystems. Values are mean ± standard error.

The REE concentration in *A. marina* leaves was not significant (*t*-test; *p* > 0.05) between the two mangrove ecosystems assessed (Table 1). However, a similar pattern of REE distribution in *A. marina* leaves and mangrove sediment was established, with higher concentrations in Jazan mangrove leaves. The higher sum total of REEs in *A. marina* leaves from the Jazan mangrove was about 1/18 of the total sum in sediment, while the lowest value for *A. marina* leaves in the Al Wajah mangrove was about 1/20 of the sum total in sediment (Table 1). This indicates that the **∑**REE in the Al Wajah and Jazan mangrove sediments were 20- and 18-fold that of the **∑**REE in their *A. marina* leaves, respectively. This is supported by the distribution of REEs in sediment and leaves presented in Figure 4, with higher distributions in sediment than leaves and in the Jazan and Al Wajah mangrove ecosystems. For specific REEs in *A. marina* leaves and sediment, the heat map shows that all the concentrations of the 14 REEs determined in *A. marina* leaves are associated (0.0–0.5) with the concentrations in the sediment (Figure 5). However, the BCF values are less than 1 for all the REEs determined; the recorded BCF values for Lu (0.33) and Tm (0.32) were the highest, while the lowest BCF recorded was for Nd (0.09) (Figure 6).

## 3. Discussion

### 3.1. Influence of Sediment Grain Size on REE Concentrations and Fractionation

Studies indicate that sediment grain size type is a vital factor for REE accumulation in sediments from the Al Wajah and Jazan mangrove ecosystems; this was supported by results from the two mangroves subjected to data analysis. Aquatic ecosystems encompass a combination of diverse products of physicochemical processes functioning in various aspects of drainage basins [18]. This could be linked to or support the influence of grain size types on elements concentrations and distribution, which has also been reported previously to partly reflect the impacts of the hydrodynamic environment [19]. It is important to note that transportation, resuspension, and deposition of allochthonous sediments into an aquatic environment such as the mangrove ecosystem could be influenced by hydrodynamics [19,20]. However, the fractionation of REEs is largely due to mineralogical controls and the diversity of sediment detrital minerals composed of different grain size types triggered by the complex nature of patterns for drainage weathering and hydrodynamic sorting [21,22]. In other studies, different minerals were reported to compose specific REE characteristics due to different grain size types, and differentiation in sizes during transportation and sediment deposition caused some level of differentiation in mineralogy [18,21].

Fine-grain particles have large surface area as one of their properties that can influence an increase in element sorption [23,24]. As such, it is reasonable to hypothesize that there exists a positive correlation between clay silt sediment particles and concentrations of REEs. This could constitute a reason for or enable a better understanding of the correlation between the sediment grain size types and concentrations of REEs in this study [25,26]. Variations in metal concentrations and distribution and their relationship with sediment grain size types have been reported elsewhere, and notably, these studies demonstrated that fine-grain sediment in mangroves possess the capacity for adsorption of REEs [27,28].

The Jazan mangrove is a coastal area open to the public with lots of industrial activities and other anthropogenic activities, and this together with changes in sediment nature (grain size) could form a major reason for higher concentrations of REEs [12,29]. Notably, **∑**REE in Jazan mangrove sediment was almost two-fold that of the **∑**REE determined in Al Wajah mangrove sediment, which is a natural reserve area with rare or few anthropogenic activities. Elsewhere, in the assessment of REEs in six mangrove ecosystems in the central Red Sea [19], the average of **∑**REE (42.56 mg/kg) was lower than (about 1/3 of) the value reported from Jazan mangrove (112.54 mg/kg) in this study was established. In another study on the Egyptian coast of the Red Sea [30], the **∑**REE (47.55 mg/kg) reported was about 1/2 of the **∑**REE (112.54 mg/kg) in the Jazan mangrove ecosystem. The depletion of HREEs relative to the lighter ones observed in this study is similar to previous observations elsewhere on the assessment of REEs in mangroves and mangrove soil profiles [11,24,31]. HREE depletion in benthic sediment could be as a result of their greater tendency towards the formation of soluble carbonates in a stable state and complexes of organic forms with ligands than the lighter REEs (LREE and MREE) [32]. HREEs have a high tendency towards being less reactive than LREEs and MREEs, and are well adapted or linked with phases in solid states because they have more pronounced complexation together with ligands located on surfaces of colloids and particles [19,33]. The removal and/or preference of LREEs could also be based on this phenomenon [11].

### 3.2. REE Fractionation and Sediment Quality Index (Igeo)

The utilization of Post-Archean Australian Shale (PAAS) [34] in the normalization of REE concentrations in sediments of marine ecosystems is vast and quite vital in revealing the comparative fractionation of REEs and the source of REEs, and enables an easy comparison of various findings in ecosystems. The results of REE fractionation are widely utilized as tracers for the determination of the effect of contamination sources and mangrove sediment on flora and fauna diversity, and determination of the chemistry of the environment [8,19].

The use of REE fractionation in the determination of detailed LREE, MREE, and HREE enrichment is vital in the assessment of REEs in an ecosystem, even though direct computation using the sum of the concentrations is measured initially. It is important to note that (La/Yb)n, (Sm/La)n, and (Yb/Sm)n fractions of Post-Archean Australian Shale (PAAS) normalized values were used as a model for REE fractionation and represent LREEs, MREEs, and HREEs, respectively [12,24]. Higher fractions of PAAS normalized (Sm/La)n were recorded in Al Wajah (1.96) and Jazan (2.17) compared to (Yb/Sm)n, and the average (Sm/La)n (2.07) being higher than the average (Yb/Sm)n (1.08) is an indication of the predominance of lighter REEs (LREEs and MREEs) over HREEs in sediments, and more precisely, the levels of lighter REEs (LREEs and MREEs) in Jazan are higher than those of the Al Wajah mangrove. However, the higher fraction (La/Yb)n (0.49) at Al Wajah than (La/Yb)n (0.41) is an indication of the predominance of LREEs at Al Wajah, while MREEs and HREEs were predominant at the Jazan mangrove, as reflected by the higher values of (Sm/La)n (2.17) and (Yb/Sm)n (1.13).

Fractionation of REEs has also been defined by some authors using a scale of 1; a ratio equal to 1 was stated to be an indication of no fraction, whereas a ratio < 1 indicates depletion, and a ratio > 1 implies enrichment of REEs [12,35]. In support of the aforementioned, multi-elemental ratios R(M/L) and R(H/M) with positive and negative values signify fractionation patterns enriched with lighter rare earth elements (MREEs and LREEs), and depletion of HREEs [36,37]. In another study, in the Pichavaram mangrove ecosystem, similar findings were reported on REE fractionation, with an insight into higher concentrations of lighter REEs being linked to clay-silt sediment composition in the mangrove ecosystem [37,38]. This was supported by the positive correlation (r = 0.58) between the clay silt grain size type and the lighter REEs. Nonetheless, it is critical to note that deposition of sediment in a high-sediment regime and rapid burial could lead to a reduced time frame of exposure to REEs in dissolved form with sediment. This causes a restriction in the capacity of adsorption of the sediment and the possibility of REE depletion, leading to dissimilarities in concentrations of REEs in mangrove sediment [11,37].

Geo-accumulation index (Igeo) is an important sediment quality index used for the determination of the extent of contamination of metal and the role of human activities in sediment metal accumulation [29,39,40]. Anthropogenic sources such as industrialization and chemicals or substances from anthropogenic activities such as pesticides and fertilizers contained in agricultural waste could form the key reason for strong to extreme contamination (4 ≤ Igeo ≥ 5) of certain REEs determined in this study [41,42,43,44]. Notably, REEs are often used as fertilizers, which is a direct approach or application to plant/sediment interphase for the purposes of growth, yield, and quality improvement. However, this process or usage of REEs could increase their concentration and sediment or soil contamination [9].

The use of the various applications in several technologies for different materials’ production and finished products involves the exploitation of REEs; this has led to pristine ecosystem contamination as a result of poor methods of industrial effluent disposal [9,19]. Dy, Er, and Yb might have originated primarily from anthropogenic activities and crustal material due to their moderate to strong (1 ≤ Igeo ≥ 3) contamination of the sediment. Conversely, the negative Igeo (Igeo < 0) values for Eu, Tb, Ho, Tm, and Lu suggest possible sources of these REEs to be local and natural [19,45]. Similarly, in China, high REE contamination was linked to anthropogenic activities such as industrial activities, with iron and steel smelting as the major activities [29,46].

### 3.3. Bio-Concentration Factor (BCF) and REE in Avicennia Marina

Bioaccumulation of metals in plants due to the interaction between plants and sediment is commonly evaluated using Bio-Concentration Factor (BCF) [19,24]. The values for BCFs for all the REES determined in this study were less than 1 (BCFs < 1); this either signifies hypo-accumulation of the REEs by *A. marina* mangrove or the role of an effective mechanism that is involved in detoxification or exclusion of chemicals in *A. marina* [19,47]. Elsewhere, in islands of Indian Sundarban and some mangroves stands in the central Red Sea, results of BCFs reported from these mangroves were in line with our findings with BCFs for REEs less than 1 (BCFs < 1). However, the highest BCF (0.33) determined in this present study was almost three-fold that of the highest value of BCF (0.10) established in Indian Sundarban [12], and 0.01 times greater than the highest BCFs previously determined in six mangroves from the central Red Sea [19].

The composition of bioavailability forms of sediment REEs and the presence of an efficient mechanism or capacity for REE uptake in the plant can significantly affect the phytoextraction of REEs [19,48]. The major reason for the substantial dissimilarity in REE composition in *A. marina* leaves could be due to the bioavailable REE content in the two mangroves investigated. Previous studies have established a positive correlation between increased REE phytoextraction, the concentration of REE in sediment, and environmental variations due to anthropogenic influence or weathering of chemical nature, with the tendency of affecting REE sequestration [19,49].

## 4. Materials and Methods

### 4.1. Study Area 

**Figure 7:**
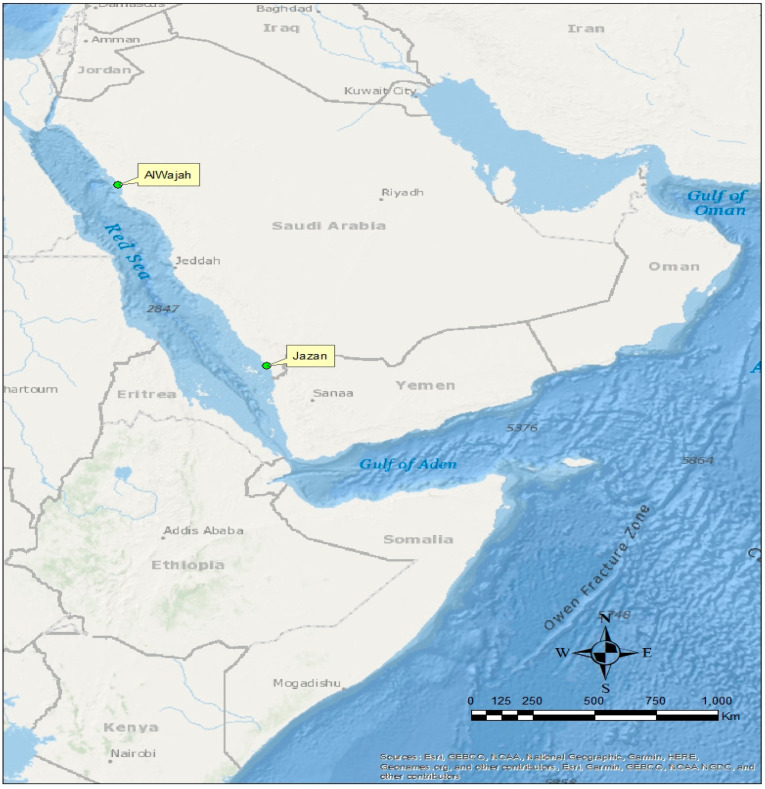
Map showing Al Wajah and Jazan mangrove ecosystems in northern and southern Central Red Sea.

The Red Sea of Saudi Arabia encompasses an area of mangroves of approximately 135 Km^2^, and the mangroves are disseminated to the northern boundary at 28.207302° N [50]. The arid environment with high temperatures and sparse rainfall is associated with the central Red Sea. In the central Red Sea of the kingdom of Saudi Arabia, some mangrove habitats appear as a narrow fringe supporting halophytes. These mangroves could at times be flooded [26,51]. The abundance of mangroves and levels of anthropogenic activities were used as criteria in the selection of the mangrove ecosystems investigated. Two (2) mangrove ecosystems in Jazan and AlWajah (Figure 7) were selected to accomplish our objectives. In the Jazan mangrove (16°56′38.3″ N, 42°32′31.9″ E), there are various anthropogenic activities, with less control in the catchment, while the Al Wajah mangrove (25°18′25.9″ N, 37°06′51.1″ E) is a reserved area with fewer anthropogenic activities and control of such activities. These two mangrove ecosystems are dominated by monospecific stands of the *Avicennia marina*.

### 4.2. Sampling and Determination of REE

Thirty (30) samples in each mangrove ecosystem (a total of 60 samples) were collected from two mangrove ecosystems in the northern and southern central Red Sea, Saudi Arabia. *A. marina* leaves and mangrove surface sediments between 0–20 cm were sampled twice monthly from May 2020 to April 2021 from Jazan and Alwajah mangrove ecosystems. At the time of sample collection, variation in the water depth was from 1 to 11 m. For each ecological unit, 15 sediment samples and leaf samples each from 15 mangrove trees were collected in replicate. Van Veen grab-250 cm^2^ was utilized in the collection of sediment samples, which were kept in zip lock bags inside an icebox to be transferred to the laboratory for further analyses. For sediment samples, 0.4 g was weighed into a digestion vessel of 50 mL capacity, and 8 mL HNO_3_:HCl (1:1) for acid digestion was added. An Anton-Paar PE Multiwave 3000 microwave oven was then used for the digestion of samples at 200 °C for 1 h [52]. Digested samples were kept in a volumetric flask at room temperature, and topped up to 50 mL with Ultrapure Millipore Q water, shaken, and allowed to sit overnight. Filtration of the solution was done using a GF/F filter (Whatman), which was then analyzed for concentrations of rare earth elements (La, Ce, Pr, Nd, Sm, Eu, Gd, Tb, Dy, Ho, Er, Tm, Yb, and Lu) using an Agilent 7700× dual pump Inductively Coupled Plasma-Mass Spectrometer (ICP-MS) [53].

For leaf samples, samples were cleaned using deionized water. Both leaf and sediment samples were dried in an oven at 40–45 °C for 48 and then crushed into powder form with agate mortar and pestle and sieved through 53 µm nylon mesh. The leaf sample was acid digested in HNO_3_:H_2_O_2_ (3:1) at 180 °C for 45 min using 0.2 g of the sieved samples. The formation of calibration curves was achieved by analyzing standard mixture solutions comprising 14 elements at concentrations of 0.5, 1, 5, 10, 20, 50, and 100 µg/L, with 0.999 linear fitting rates. The analytical method for quality control was assessed using standard reference materials GSS-1 and GSV-2 for sediments and leaves, respectively. To confirm repeatability and sensitivity, the solutions of known concentrations used as standard solutions were placed into the sequence of samples for every eight samples. The repossessions of REEs in percentage from the accuracy of the analytical method ranged from 92.68–103.21% and 81.82–116.67% for sediment and leaves, respectively (Table S1, Supplementary Materials). The acceptance of analytical precision and accuracy was based on when the standard deviation was <5% for the rare earth elements from the results of the replications of measurements of the samples and standards.

### 4.3. Grain Size Analysis in Sediment

The total weight of the oven-dried sediment was measured. Fragmentation of solidified aggregates was done by soaking the dried sediments in distilled water for 24 h. The sediments were washed and separated into fractions of gravel (>2 mm), coarse grain (0.063~2 mm), and mud (clay and silt, <0.063 mm) after passing through 0.063 mm and 2 mm sieves. The computation of percentages of sediment grain sizes relative to the total weight was achieved after the fractions from the residue were dried at 40 °C and weighed [26,38,54].

### 4.4. Sediment Quality Index and Bio-Concentration Factor (BCF)

The geo-accumulation index based on seven enrichment classes (Table S2, Supplementary Materials) [55] was utilized as the sediment quality index, and was used to measure REE contamination levels in the sediment of the two mangroves investigated using the following formula:

(1) $$I_{geo}=log_{2\;}\left[\frac{C_{n}}{1.5\times B_{n}}\right]$$

where *C_n_* = Concentration of a particular REE in the sediment, B_n_ = Geochemical background level of that REE obtained for sedimentary rocks (Shales) as described by Turekian and Wedepohl [56], and 1.5 = Correction factor [57] to reduce the effect of variations as a result of sediment lithology.

The bioavailability of REE in *A. marina* was determined using the bio-concentration factor (BCF) to reveal the efficiency of the mangrove to accumulate REEs using the following formula:

(2) $$BCF=\frac{C_{leaves}}{C_{sediment}}$$

where *C_leaf_* and *C_sediment_* = Concentrations of a given REE in leaves and sediment, respectively.

### 4.5. Data Analyses

The Student’s *t*-test was used for comparison between mean REE concentrations in sediments, leaves, BCF, Igeo, and sediment grain sizes of the two mangrove ecosystems. Principal component analysis (PCA) biplot and loadings were used to determine the relationship between sediment grain sizes and REE concentrations in sediments, while a heat map was used to determine the relationship between REEs in sediment and *A. marina* leaves. The data were analyzed using R for Windows (v. 4.0.3).

The pattern of distribution and REE bioavailability were categorized using fraction ratios (La/Yb, Sm/La, and Yb/Sm) after the concentrations of REE were normalized (*n*) to the Post-Archean Australian Shale (PAAS) [34]. The calculation of multi-elemental ratios was done as described by Duvert et al. [36] and Noack et al. [58] using the formulas below:

(3) R(ML)=logMREEnLREEn=log[(Gdn+Tbn+Dyn)3(Lan+Prn+Ndn)3]

(4) R(HM)=logHREEnMREEn=log[(Tmn+Ybn+Lun)3(Gdn+Tbn+Dyn)3]

where *R*_(*M/L*)_ = Ratio between medium and light REEs, R_(H/M)_ = Ratio between heavy and medium REEs, and *n* refers to PAAS-normalized concentrations.

The non-inclusion of Ce and Eu in the formulas is because of their potential to exhibit an oxidation state. However, a geometric method was used to compute the anomalies of Ce and Eu; this was achieved by assuming that the closest neighboring elements act linearly on log-linear plots [36,59]. The formulas below were used to compute the anomalies:

(5) $$\delta Ce=\frac{2Ce_{n}}{La_{n}+Pr_{n}}$$

(6) $$\delta Eu=\frac{2Eu_{n}}{Sm_{n}+Gd_{n}}$$

where *δCe* and *δEu* are the measure of the anomalies for Ce and Eu, and *n* refers to PAAS normalized concentrations.

## 5. Conclusions

Fractionating causes a significant enrichment of lighter REEs over HREEs in Al Wajah and Jazan mangroves. This is supported by positive and negative multi-elemental ratios R(M/L) and R(H/M), and the enrichment of lighter REEs over the HREEs is attributed to HREEs having a high tendency towards being less reactive than LREEs and MREEs, and the preference for removal of the lighter REEs. However, a higher fraction (La/Yb)n (0.49) at Al Wajah than (La/Yb)n (0.41) is an indication of the predominance of LREEs at Al Wajah, while MREEs and HREEs were predominant at the Jazan mangrove, as reflected by the higher values of (Sm/La)n (2.17) and (Yb/Sm)n (1.13). In addition, the anomalies of Eu were negative for the two mangroves investigated, possibly as a result of dominant reducing conditions in mangrove sediments.

The REE concentrations in Al Wajah and Jazan mangrove ecosystems were significant, with higher concentrations in the Jazan mangrove ecosystem. Different anthropogenic impacts in these two mangroves could form the key reasons for the differences recorded. Clay silt sediment grain size type influences increase REE concentration; however, BCF reveals hypo-accumulation potential or capacity of REE by *A. marina*, with similarity in REE distribution patterns in sediment and *A. marina*. In addition, using the scale of Igeo, based on six classes of classification of the level of contamination, the mangrove sediments were not significantly different and were strong to extremely contaminated with La, Ce, Pr, Nd, Sm, and Gd, and moderately to strongly contaminated with Dy, Er, and Yb, but not contaminated (<0) with Eu, Tb, Ho, Tm, or Lu, showing negative Igeo values. In addition, BCFs for all the REEs determined in this study signify hypo-accumulation of the REEs by *A. marina* mangrove or the role of an effective mechanism that is involved in detoxification or exclusion of chemicals in *A. marina*. There is a dire need for periodic monitoring of REE concentrations in the mangroves investigated, especially the Jazan mangrove ecosystem. This is to keep track of sources of this metal contamination and to develop strategies for control and conservation of these important ecosystems.

## Figures and Tables

**Table 1:** Rear earth elements’ composition (mg/kg) and fractions in northern and southern central Red Sea mangroves.

	AlWajah	Jazan				AlWajah	Jazan		
Sediment			Average	*p*-Value	Leaves			Average	*p*-Value
La	12.67 ± 1.73	17.95 ± 2.32	15.31 ± 2.64	0.007	La	0.70 ± 0.11	0.86 ± 0.12	0.78 ± 0.08	0.887
Ce	28.59 ± 2.68	38.35 ± 4.46	33.47 ± 4.88	0.004	Ce	1.48 ± 0.13	1.64 ± 0.20	1.56 ± 0.06	0.776
Pr	3.61 ± 0.23	5.73 ± 0.12	4.67 ± 1.06	0.001	Pr	0.18 ± 0.02	0.34 ± 0.01	0.26 ± 0.07	0.766
Nd	16.62 ± 2.14	22.60 ± 3.59	19.61 ± 2.99	0.002	Nd	0.63 ± 0.09	0.79 ± 0.03	0.71 ± 0.06	0.775
Sm	3.62 ± 0.29	5.65 ± 0.19	4.64 ± 1.02	0.020	Sm	0.17 ± 0.01	0.32 ± 0.04	0.25 ± 0.05	0.895
Eu	0.96 ± 0.05	2.34 ± 0.06	1.65 ± 0.69	0.318	Eu	0.06 ± 0.01	0.21 ± 0.01	0.14 ± 0.05	0.448
Gd	3.43 ± 0.30	5.45 ± 0.21	4.44 ± 1.01	0.449	Gd	0.15 ± 0.02	0.32 ± 0.04	0.24 ± 0.07	0.669
Tb	0.58 ± 0.04	0.89 ± 0.03	0.74 ± 0.16	0.496	Tb	0.05 ± 0.01	0.21 ± 0.01	0.13 ± 0.07	0.768
Dy	3.32 ± 0.31	5.09 ± 0.06	4.21 ± 0.89	0.001	Dy	0.12 ± 0.02	0.28 ± 0.02	0.20 ± 0.06	0.557
Ho	0.66 ± 0.07	0.94 ± 0.02	0.80 ± 0.14	0.004	Ho	0.05 ± 0.01	0.22 ± 0.01	0.14 ± 0.09	0.975
Er	1.92 ± 0.20	3.27 ± 0.02	2.60 ± 0.68	0.001	Er	0.09 ± 0.02	0.24 ± 0.01	0.17 ± 0.04	0.856
Tm	0.32 ± 0.03	0.52 ± 0.01	0.42 ± 0.10	0.128	Tm	0.05 ± 0.01	0.24 ± 0.01	0.15 ± 0.03	0.368
Yb	1.89 ± 0.20	3.25 ± 0.02	2.57 ± 0.68	0.001	Yb	0.07 ± 0.02	0.22 ± 0.01	0.15 ± 0.08	0.697
Lu	0.29 ± 0.02	0.51 ± 0.01	0.40 ± 0.11	0.136	Lu	0.05 ± 0.01	0.21 ± 0.01	0.13 ± 0.04	0.849
∑REE	78.47 ± 7.89	112.54 ± 10.48	95.51 ± 17.04	0.001	-	3.84 ± 0.76	5.94 ± 0.91	4.98 ± 0.22	0.568
(La/Yb)n	0.49 ± 0.02	0.41 ± 0.01	0.45 ± 0.04	0.008	-	-	-	-	-
(Sm/La)n	1.96 ± 0.04	2.17 ± 0.02	2.07 ± 0.12	0.005	-	-	-	-	-
(Yb/Sm)n	1.03 ± 0.01	1.13 ± 0.01	1.08 ± 0.05	0.006	-	-	-	-	-
R(M/L)	0.25 ± 0.01	0.28 ± 0.03	0.27 ± 0.12	0.043	-	-	-	-	-
R(H/M)	−0.02 ± 0.01	0.030 ± 0.001	0.005 ± 0.030	0.004	-	-	-	-	-
δCe	0.97 ± 0.11	0.86 ± 0.09	0.92 ± 0.06	0.023	-	-	-	-	-
δEu	1.27 ± 0.46	1.98 ± 0.39	1.63 ± 0.36	0.015	-	-	-	-	-

The values in the table are mean ± standard error.

## Data Availability

Data can be shared upon personal request to the authors.

## Supplementary Materials

The following supporting information can be downloaded at https://www.mdpi.com/article/10.3390/molecules27144335/s1. Table S1: Analytical results achieved on certified reference materials for sediment and leaves; Table S2: Classification of Sediment quality (Geo-Accumulation Index).

## Sample Availability

Samples of the compounds studied are available from the authors.
