# Supplementary material for: Rare Earth Elements and Bioavailability in Northern and Southern Central Red Sea Mangroves, Saudi Arabia
Source: Molecules. 2022 Jul 6;27(14):4335. doi: 10.3390/molecules27144335 (PMC9318687; doi:10.3390/molecules27144335)
Supplement: Supplementary file 1 [file molecules-27-04335-s001.zip › molecules-1784404-supplementary.pdf]

Supplementary Material

Supplementary Tables

Table S1. Analytical results achieved on certified reference materials for sediment and leaves.

Sediment	Elements (mg/kg)	GSS-1			Leaves	GSV-2		
		Certified	Experimental	Recovery (%)		Certified	Experimental	Recovery (%)
	**La**	34.00 ± 4.47	30.20 ± 4.21	88.82		1.25 ± 0.02	1.23 ± 0.11	91.11
	**Ce**	70.00 ± 17.11	66.80 ± 7.22	95.43		2.20 ± 0.01	2.24 ± 0.92	101.82
	**Pr**	7.50 ± 0.25	7.45 ± 0.52	99.33		0.29 ± 0.04	0.30 ± 0.03	103.45
	**Nd**	28.00 ± 1.22	28.90 ± 3.02	103.21		1.00 ± 0.03	0.95 ± 0.02	95.00
	**Sm**	5.20 ± 0.33	5.13 ± 0.81	98.65		0.19 ± 0.01	0.21 ± 0.04	110.53
	**Eu**	1.00 ± 0.01	0.98 ± 0.02	98.00		0.45 ± 0.02	0.51 ± 0.03	113.33
	**Gd**	4.60 ± 0.94	4.61 ± 0.04	100.22		0.17 ± 0.02	0.18 ± 0.03	105.88
	**Tb**	0.75 ± 0.03	0.71 ± 0.03	94.67		0.29 ± 0.001	0.33 ± 0.02	113.79
	**Dy**	4.60 ± 0.08	4.33 ± 0.21	94.13		0.14 ± 0.02	0.16 ± 0.04	114.29
	**Ho**	0.87 ± 0.02	0.84 ± 0.05	96.55		0.13 ± 0.002	0.14 ± 0.02	107.69
	**Er**	2.60 ± 0.15	2.52 ± 0.43	96.92		0.08 ± 0.004	0.09 ± 0.01	112.50
	**Tm**	0.42 ± 0.02	0.43 ± 0.02	102.38		0.10 ± 0.002	0.11 ± 0.01	110.00
	**Yb**	2.70 ± 0.33	2.55 ± 0.03	94.44		0.06 ± 0.002	0.07 ± 0.004	116.67
	Lu	0.41 ± 0.02	0.38 ± 0.01	92.68		0.11 ± 0.001	0.09 ± 0.001	81.82

Table S2. Classification of Sediment quality (Geo – accumulation Index).

I_{geo} Value	I_{geo} Class	Sediment Quality
<0	0	Uncontaminated
0–1	1	Uncontaminated to moderately contaminated
1–2	2	Moderately contaminated
2–3	3	Moderately to strongly contaminated
3–4	4	Strongly contaminated
4–5	5	Strongly to extremely contaminated
>5	6	Extremely contaminated
